# Limits of Clinical Restaging in Detecting Responders After Neoadjuvant Therapies for Rectal Cancer

**DOI:** 10.1097/DCR.0000000000002450

**Published:** 2022-11-16

**Authors:** Simona Deidda, Gaya Spolverato, Giulia Capelli, Riccardo Quoc Bao, Lorenzo Bettoni, Filippo Crimì, Luigi Zorcolo, Salvatore Pucciarelli, Angelo Restivo

**Affiliations:** 1 Department of Surgical Science, University of Cagliari, Cagliari, Italy; 2 Department of Surgical, Oncological and Gastroenterological Sciences, University of Padova, Padua, Italy; 3 Department of Medicine (DIMED), Institute of Radiology, University of Padova, Padua, Italy

**Keywords:** Accuracy, Clinical response, Neoadjuvant therapy, Rectal cancer

## Abstract

**BACKGROUND:**

Accurate clinical restaging is required to select patients who respond to neoadjuvant chemoradiotherapy for locally advanced rectal cancer and who may benefit from an organ preservation strategy.

**OBJECTIVE:**

The purpose of this study was to review our experience with the clinical restaging of rectal cancer after neoadjuvant therapy to assess its accuracy in detecting major and pathological complete response to treatment.

**DESIGN:**

This was a retrospective cohort study.

**SETTING:**

This study was conducted at 2 high-volume Italian centers for Colorectal Surgery.

**PATIENTS:**

Data were included from all consecutive patients who underwent neoadjuvant therapy and surgery for locally advanced rectal cancer from January 2012 to July 2020. Criteria to define clinical response were no palpable mass, a superficial ulcer <2 cm (major response), or no mucosal abnormality (complete response) at endoscopy and no metastatic nodes at MRI.

**MAIN OUTCOME MEASURES:**

The main outcome measures were sensitivity, specificity, positive predictive values, and negative predictive values of clinical restaging in detecting pathological complete response (ypT0) or major pathological response (ypT0-1) after neoadjuvant therapy.

**RESULTS:**

A total of 333 patients were included; 81 (24.3%) had a complete response whereas 115 (34.5%) had a pathological major response. Accuracy for clinical complete response was 80.8% and for major clinical response was 72.9%. Sensitivity was low for both clinical complete response (37.5%) in detecting ypT0 and clinical major response (59.3%) in detecting ypT0-1. Positive predictive value was 68.2% for ypT0 and 60.4% for ypT0-1.

**LIMITATIONS:**

The main limitation of the study its retrospective nature.

**CONCLUSION:**

Accuracy of actual clinical criteria to define pathological complete response or pathological major response is poor. Failure to achieve good sensitivity and precision is a major limiting factor in the clinical setting. Current clinical assessments need to be revised to account for indications for rectal preservation after neoadjuvant chemoradiotherapy. See **Video Abstract** at http://links.lww.com/DCR/C63.

**LÍMITES DE LA REESTADIFICACIÓN CLÍNICA EN LA DETECCIÓN DE RESPONDEDORES DESPUÉS DE TERAPIAS NEOADYUVANTES PARA EL CÁNCER DE RECTO:**

**ANTECEDENTES:**

Se requiere una nueva reestadificación clínica precisa para seleccionar pacientes que respondan a la quimiorradioterapia neoadyuvante para el cáncer de recto localmente avanzado y que puedan beneficiarse de una estrategia de preservación de órganos.

**OBJETIVO:**

El propósito de este estudio fue revisar nuestra experiencia con la reestadificación clínica del cáncer de recto después de la terapia neoadyuvante para evaluar su precisión en la detección de una respuesta patológica importante y completa al tratamiento.

**DISEÑO:**

Estudio de cohorte retrospectivo.

**AJUSTE:**

Este estudio se realizó en dos centros italianos de alto volumen para cirugía colorrectal.

**PACIENTES:**

Incluimos datos de todos los pacientes consecutivos que se sometieron a terapia neoadyuvante y cirugía por cáncer de recto localmente avanzado desde enero de 2012 hasta julio de 2020. Los criterios para definir la respuesta clínica fueron ausencia de masa palpable, úlcera superficial <2 cm (respuesta mayor) o ausencia de anomalías en la mucosa. (respuesta completa) en la endoscopia, y sin ganglios metastásicos en la resonancia magnética.

**PRINCIPALES MEDIDAS DE RESULTADO:**

Exploramos la sensibilidad, la especificidad, los valores predictivos positivos y negativos de la reestadificación clínica para detectar una respuesta patológica completa (ypT0) o mayor (ypT0-1) después de la terapia neoadyuvante.

**RESULTADOS:**

Se incluyeron 333 pacientes; 81 (24,3%) tuvieron una respuesta completa mientras que 115 (34,5%) tuvieron una respuesta patológica mayor. La precisión de la respuesta clínica completa y la respuesta clínica importante fue del 80,8 % y el 72,9 %, respectivamente. La sensibilidad fue baja tanto para la respuesta clínica completa (37,5 %) en la detección de ypT0 como para la respuesta clínica mayor (59,3 %) en la detección de ypT0-1. El valor predictivo positivo fue del 68,2 % para ypT0 y del 60,4 % para ypT0-1.

**LIMITACIONES:**

Nuestro estudio tiene como principal limitación su carácter retrospectivo.

**CONCLUSIÓNES:**

La precisión de los criterios clínicos reales para definir una respuesta patológica completa o mayor es pobre. El hecho de no lograr una buena sensibilidad y precisión es un factor limitante importante en el entorno clínico. La indicación para la preservación rectal después de la quimiorradioterapia neoadyuvante necesita una mejora de la evaluación clínica actual. Consulte **Video Resumen** en http://links.lww.com/DCR/C63. *(Traducción—Dr. Mauricio Santamaria*)

Multimodal and multidisciplinary treatment, with neoadjuvant chemoradiotherapy (CRT) followed by total mesorectal excision (TME), is the standard of care for locally advanced rectal cancer (LARC).^1,2^ This approach significantly decreases the rate of local recurrence^2,3^ and may also result in complete tumor regression in 15% to 30% of patients.^4^ For this subset of patients, there is a growing interest in the use of rectal-sparing therapeutic strategies, such as local excision or a “watch-and-wait” approach, to avoid the short- and long-term drawbacks of TME.^5–18^

Selection for a rectal-sparing strategy is made by clinical assessment (restaging) of the tumor response. In current clinical practice, endoscopy and digital rectal examination (DRE)^6,19–21^ provide the macroscopic assessment of the mucosa, and MRI allows for the analysis of deeper layers of the rectal wall and mesorectum and of possible lymph node involvement.^22,23^

Different criteria for defining the clinical complete response (cCR) and clinical major response (cMR) have been proposed^20,24^ to select patients who might benefit from organ preservation strategies. However, the accuracy and precision of the current selection criteria have been reported with mixed results.^25–28^

Accordingly, the main aim of the present study was to assess the accuracy of the current clinical assessment of the tumor response after CRT and to test its overall performance in selecting patients for a conservative therapeutic approach.

## MATERIALS AND METHODS

From January 2012 to July 2020, the data of consecutive patients with LARC who had neoadjuvant treatment and subsequent surgery were identified from prospectively maintained databases from 2 high-volume Italian referral centers for colorectal surgery.

Patients aged <18 years with histologically proven rectal adenocarcinoma and a pretreatment stage of II or III were included in the study. Patients with synchronous colorectal cancers, pretreatment stage I or IV, or concomitant familial polyposis syndrome or IBD; those who followed a watch-and-wait strategy for clinical response; and those who had undergone surgery in an emergency setting were excluded from the study.

Initial staging included a DRE, complete colonoscopy, CEA levels, and chest and abdominal CT scan. Pelvic MRI was used to assess the pretreatment T and N stage in most patients. Endorectal ultrasound images were used alternatively or in combination in selected cases of early disease or when MRI was not feasible (55 patients [16.5%]). Lymph nodes with a diameter >5 mm along the short axis at imaging were considered metastatic.^24,29^

Neoadjuvant treatment schedules might consist of long-course radiotherapy with a dose of 45 Gy administered over 5 weeks (25 fractions of 1.8 Gy/d) with a 3-field technique. Short-course radiotherapy (25 Gy in 5 fractions) with long waiting time was used if patients were unfit for long-course treatment. Factors related to this were age, disability, preexisting diseases, physical impairments, and cognitive impairments. Preoperative chemotherapy was based on the administration of 5-fluorouracil (5-FU) either in a daily oral preparation (capecitabine 1650 mg/m^2^/d) taken during the radiation period, in a bolus infusion (5-FU 325 mg/m^2^/d × 5 d) during weeks 1 and 5, or as a continuous infusion for 5 d/wk during the entire 5-week radiation period (5-FU 250 mg/m^2^/d).

Per routine protocol in both centers, all patients underwent clinical restaging 8 weeks after the end of neoadjuvant CRT.^29–32^ Clinical restaging was assessed through DRE, endoscopy, and MRI for node evaluation. MRI was also used to detect rectal wall thickness or alterations, but only lymph node status was chosen as a criterion to select for an organ-sparing strategy.^24,29^ We did not consider PET CT to assess response.^33,34^ All images were reassessed by a radiologist for each center.

A cCR was defined as no palpable mass at DRE, no mucosal abnormality at endoscopy (except for a flat scar or telangiectasia, which was not considered as a mucosal abnormality), and no metastatic nodes at MRI (<5 mm).^24,29^

A cMR was defined as the absence of mass at DRE and the presence of no more than a small mucosal irregularity or superficial ulcer within 2 cm in diameter at endoscopy and no suspicious nodes on MRI. Lymph nodes with a diameter of >5 mm along the short axis were considered metastatic.

Surgery consisted either of a surgical resection with TME surgery or a local excision with transanal endoscopic microsurgery or a transanal endoscopic operation. Local excision was proposed for those patients who had cCR or cMR, and it was considered primarily as an excisional biopsy.^24,29^ Based on histopathology, patients were recommended for subsequent TME surgery if, after local excision, the patient was found to have an adenocarcinoma >ypT1 or with either high grade, positive margins or TRG (tumor regression grade) ≥3. Senior colorectal surgeons performed all procedures from each center.

All patients were thoroughly discussed in a multidisciplinary tumor board conference before and after neoadjuvant treatment.

The accuracy of clinical restaging was explored by a correlation with a final histopathologic examination based on the eighth TNM staging system of the American Joint Committee on Cancer.^35^

Histopathology included ypT status, TRG according to the modified Mandard classification,^24^ and the involvement of margins, degree of differentiation, and presence/absence of lymphatic, perineural, or vascular invasion. A pathological complete response (pCR) was defined as a final pathological stage of ypT0N0M0 and a pathological major response (pMR) of ypT0-1N0M0.

### Statistical Analysis

SPSS Statistics 20 (IBM, Armonk, NY) and Stata 13.0 (StataCorp, College Station, TX) were used to perform statistical analyses. Continuous variables were expressed as the median and interquartile range (IQR); for frequencies, the corresponding 95% CI was calculated by the mid-p exact method.

To assess the accuracy of clinical restaging, we calculated the sensitivity, specificity, positive predictive value (PPV), and negative predictive value (NPV) for the cCR and cMR to assess a pCR (ypT0) or pMR (ypT0-1), respectively.

To assess the degree of correspondence between the clinical and histological findings, Cohen’s κ coefficient was calculated. This lies between 0 (casual correspondence) and 1 (absolute correspondence). Results for κ were interpreted according to Landis and Koch^36^ (as 0.0–0.2, poor; 0.2–0.4, fair; 0.4–0.6, moderate; 0.6–0.8, good; or 0.8–1.0, very good).

## RESULTS

### Study Population

Three hundred seventy-seven patients who had CRT and surgery for LARC were identified. After excluding 30 patients for whom clinical restaging data were not available and 14 who were offered a watch-and-wait strategy, 333 patients met the inclusion criteria and were included in the analysis. Demographics, clinical characteristics, and treatment characteristics of the included patients are summarized in Table 1. Overall, 208 patients (62.5%) were males and 125 patients (37.5%) were females, with a median age of 66 (IQR, 56–74) years. The median distance of the primary tumor from the anal verge was 6 (IQR, 4–9) cm.

**TABLE 1.** Patient’s characteristics

*Variable*	*Total (N = 333*)
Age, y, median (IQR)	66 (56–74)
Sex, n (%)	
Female	125 (37.5)
Male	208 (62.5)
Distance from the AV, cm, median (IQR)	6 (4–9)
cT, n (%)	
1	2 (0.6)
2	31 (9.3)
3	243 (72.9)
4	57 (17.1)
cN, n (%)	
0	101 (30.3)
1	150 (45.1)
2	82 (24.6)
cStage, n (%)	
2	101 (30.3)
3	231 (69.37)
Neoadjuvant radiotherapy, n (%)	
Long-course	319 (95.8)
Short-course – long waiting	14 (4.2)
Neoadjuvant chemotherapy, n (%)	
Yes	308 (92.5)
No	25 (7.5)
Interval between neoadjuvant treatment and surgery, wk, median (IQR)	13 (10–15)
Response, n (%)	
cCR	44 (13.2)
cMR	67 (20.1)
Null or partial response	222 (66.7)
Surgical procedure, n (%)	
LAR	222 (66.7)
APR	62 (18.6)
LE	49 (14.7)
CRM, n (%)	
Positive	10 (3)
ypT, n (%)	
0	81 (24.3)
1	33 (9.9)
2	69 (20.7)
3	142 (42.6)
4	8 (2.4)
ypN, n (%)	
0	206 (61.9)
1	48 (14.4)
2	30 (9)
x	49 (14.7)
ypStage, n (%)	
0	80 (24.02)
1	92 (27.6)
2	84 (25.2)
3	77 (23.1)

APR = abdominoperineal resection; AV = anal verge; cCR = clinical complete response; cMR = clinical major response; CRM = circumferential resection margin; CRT = chemoradiotherapy; IQR = interquartile range; LAR = low anterior resection; LE = local excision; ypNx = patients undergoing local excision

At baseline, 2 patients (0.6%) were staged as cT1, 31 patients (9.3%) as cT2, 243 patients (73%) as cT3, and 57 patients (17.1%) as cT4. One hundred one patients (45.1%) were assessed as cN1 and 82 patients (24.6%) as cN2.

All patients underwent neoadjuvant treatment: 319 patients (95.8%) had long-course radiotherapy, and 14 patients (4.2%) had short-course radiotherapy followed by a prolonged waiting interval. Also, 308 patients (92.5%) had preoperative chemotherapy. The median interval between the completion of preoperative treatment and the surgical procedure was 13 weeks (IQR, 10–15).

Two hundred eighty-four patients underwent surgical resection with TME: 222 (66.7%) patients underwent anterior resection, and 62 patients (18.6%) underwent abdominoperineal resection. Forty-nine patients (14.7%) were treated with local excision either by transanal endoscopic microsurgery or transanal endoscopic operation.

At the final histopathologic examination, 80 patients (24%) were staged ypT0, 33 patients (9.9%) ypT1, 69 patients (20.7%) ypT2, 142 patients (42.6%) ypT3, and 8 patients (2.4%) ypT4. Overall, the pMR was assessed in 115 patients (33.7%). The node status was ypN0 in 206 patients (72.5%), ypN1 in 48 patients (16.9%), and ypN2 in 30 patients (10.6%).

### Relationship Between Clinical and Pathologic Response to Neoadjuvant Treatment

Complete data on the correlation between clinical and pathological responses are shown in Table 2. Preoperative clinical assessment of response showed that 222 patients (66.7%) had no response or partial response to CRT, 67 patients (20.1%) had a cMR, and 44 patients (13.2%) had a cCR.

**TABLE 2.** Performance of restaging at predicting ypT0 and ypT0-1

		*Pathological stage (ypT0*)								
Clinical stage		*Yes*	*No*	Total	*Sensitivity % (95% CI*)	*Specificity % (95% CI*)	*PPV % (95% CI*)	*NPV % (95% CI*)	*Accuracy % (95% CI*)	*Cohen’s* κ
cCR	Yes	30	14	44	37.5 (26.9–49)	94.5 (90.9–96.9)	68.2 (54.5–79.3)	82.3 (80.1–85)	80.8 (76.1–84.9)	0.38
	No	50	239	289						
		80	253	333						
		*Pathological stage (ypT0-1*)								
		*Yes*	*No*	*Total*						
cCR	Yes	34	10	44	30.1 (21.8–39.4)	95.5 (91.8–97.8)	77.3 (63.6–86.9)	72.7 (70.1–75.1)	73.3 (68.2–77.9)	0.3
	No	79	210	289						
		113	220	333						
		*Pathological stage (ypT0-1*)								
		*Yes*	*No*	*Total*						
cMR	Yes	67	44	111	59.3 (49.6–68.4)	80 (74.1–85.1)	60.4 (52.9–67.4)	79.3 (75.2–82.8)	73 (67.9–77.7)	0.39
	No	46	176	222						
		113	220	333						
		*Pathological stage (ypT0*)								
		*Yes*	*No*	*Total*						
cMR	Yes	58	53	111	72.5 (61.4–81.9)	79.1 (73.5–83.9)	52.3 (45.4–59)	90.1 (86.4–92.9)	77.5 (72.6–81.9)	0.45
	No	22	200	222						
		80	253	333						

cCR = clinical complete response; cMR = clinical major response; NPV = negative predictive value; PPV = positive predictive value.

A significant portion of the patients who did not show a pMR or a pCR were understaged at clinical evaluation. This occurred in 14 patients (31.8%) with cCR (ypT1, n = 4 [1.6%]; ypT2, n = 6 [2.4%]; ypT3, n = 3 [1.2%]; ypT4, n = 1 [0.4%]) and in 44 patients (39.6%) with cMR (ypT2, n = 15 [6.8%]; ypT3, n = 27 [12.3%]; ypT4, n = 2 [0.9%]). Of the 80 patients with ypT0 tumors and 113 patients with ypT0-1 tumors, 50 (62.5%) and 46 (40.7%) were overstaged at clinical evaluation.

The sensitivity in detecting ypT0 was low, at 37.5% (95% CI, 26.9–49). The clinical criteria for cCR had a precision (PPV) in indicating a true complete response of almost 70% [68.2% (95% CI, 54.5–79.3)]. The overall accuracy was 80.8% (95% CI, 76.1–84.9), and Cohen’s κ coefficient was 0.38.

The sensitivity of the cMR criteria in detecting major response was significantly higher (59.3% [95% CI, 49.6–68.4]) than that for the cCR and pCR. The precision for pMR detection was, instead, lower, at 60.4% (95% CI, 52.9–67.4). The accuracy was 73% (95% CI, 67.9–77.7), and Cohen’s κ coefficient was very similar at 0.39.

In a post hoc analysis, we explored the accuracy we would have had if we had applied less strict clinical criteria for detecting the pCR by considering the correlation between a cMR and pCR. In this case, the sensitivity was 72.5% (95% CI, 61.4–81.9). However, precision was just 52.3% (95% CI, 45.4–59)]. The accuracy was 77.5% (95% CI, 72.6–81.9), and Cohen’s κ coefficient was 0.45.

**FIGURE 1.**
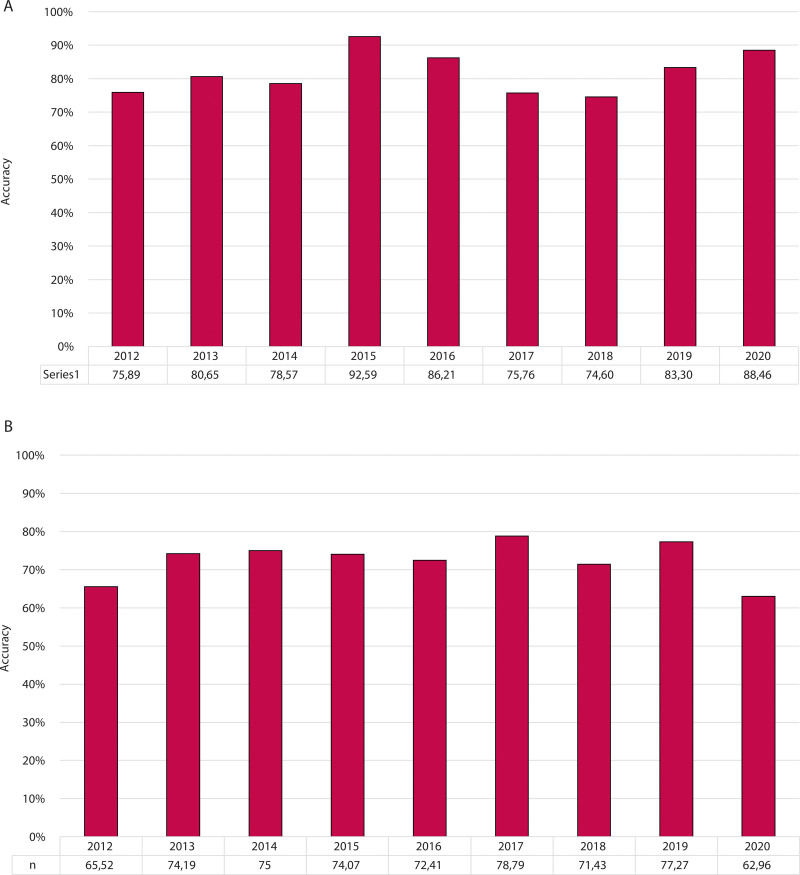
Accuracy of clinical restaging during the study period in detecting A, pathological complete response and B, major pathological response.

As shown in Figure 1, a time trend analysis showed that the accuracy of clinical and radiological assessment did not improve during the study period in detecting both pCR and pMR.

## DISCUSSION

The purpose of this study was to assess the ability of current clinical and radiological criteria to predict the pCR and pMR in patients with LARC who underwent neoadjuvant treatments.

The performance of actual clinical criteria in correctly predicting the pathological tumor response after CRT appears suboptimal. We found a just “fair” concordance coefficient (Cohen’s κ) for both complete and major response assessments. The sensitivity, especially for pCR detection, was <40%, meaning that the majority of patients with a pCR could not be detected. As was predictable, this percentage was higher with less strict clinical criteria, achieving 60% in pMR assessment, but at the expense of precision (reduced by 10%).

However, as with every medical diagnostic tool, test performance has to be judged in the context of its clinical application and utility. Considering that a pCR occurs in 15% to 30% of patients after neoadjuvant treatments,^4^ there is an increasing interest in individualizing treatment after CRT to provide less aggressive management for patients with significant downstaging. Rectum-sparing approaches, such as local excision and a “watch-and-wait” approach,^5,17,37^ can be proposed to a well-selected group of patients for whom immediate radical surgery may be delayed or avoided. However, these strategies demand a reliable method of identifying patients with a cCR or cMR. When pursuing a rectal-sparing approach, the low sensitivity reported for our definition of cCR may be a concern. Overstaging may ultimately result in a high rate of “unnecessary” surgical resections. In fact, the majority of patients (62.5%) eventually downstaged to ypT0 did not actually qualify as having a cCR in our study.

One of the major problems in actual preoperative evaluation is the lack of uniform definitions of responses. Moreover, the treatment itself can cause important changes in the structure of the rectal wall, making it difficult to differentiate fibrosis because of CRT from residual tumor.^38^

Based on criteria very similar to ours, Habr-Gama et al^21^ proposed that cCR should be identified by a complete disappearance of the tumor at endoscopy that might leave a whitening of the mucosa, with or without telangiectasia, or a complete normalization of the mucosa. However, other authors^27,39,40^ already showed that some pCRs could still have residual mucosal abnormalities such as ulcers and exophytic or nodular lesions. In a retrospective study from the Cleveland Clinic,^27^ 74% of patients with a final pCR had some residual mucosal abnormality that would have led to their being categorized as having incomplete clinical responses. Even when the clinical criteria (DRE and endoscopy) were associated with radiological imaging (endorectal ultrasound and MRI), the sensitivity seemed low, as 75% of patients with a pCR may still present with a thickness of the rectal lumen or lymph nodes in the mesorectum on MRI or ultrasound imaging.^18,22,28,41–44^

In our data, when considering the clinical criteria for cMR, the sensitivity was higher for detecting a pMR or pCR. In particular, in the diagnosis of a pCR, the criteria for cMR achieved the best overall performance, considering the higher κ coefficient and the best equilibrium between sensitivity and specificity. Less strict criteria may indeed improve selection by identifying more pCRs and thus theoretically improving the rate of rectal-sparing treatments. However, this may come at the cost of a significantly higher false-positive rate, and it could be as high as 47.7%.

From a practical point of view, a high false-positive rate could lead to an increased risk of local regrowth if one is pursuing a wait-and-see strategy. Local regrowth after this strategy has been reported in 20% to 30% of cases in major studies.^45,46^ Our data suggest that the use of less strict criteria to detect more pCRs could almost double this rate.

This consideration favors the use of local excision to achieve a good compromise between morbidity and oncological results.^47,48^

A multistep approach may allow for immediate salvage surgery if a satisfactory pathological response is not confirmed. As with different reports, local excision might be considered oncologically safe for tumors of up to ypT.^29^

It is interesting to note that the cCR criteria show the best performance in terms of precision (77%) in detecting an pMR (ypT0 or 1). In other terms, following these criteria, salvage surgery after local excision would be necessary for no more than 2 of 10 patients; thus, this would not compromise the final oncological results but would avoid major surgery in a larger number of patients.

## CONCLUSIONS

Failure to achieve good sensitivity and precision by current clinical criteria suggests that alternative methods of identifying patients who respond to CRT need to be sought. As such, newer technologies, such as systemic genetic markers, may allow a future to increase the accuracy of clinical criteria. Moreover, advanced methods such as diffusion-weighted MRI perfusion or radiomics, as well as artificial intelligence modeling, are promising, but they are currently only the subject of research.^22,33,49^

Although we believe that the findings of this study are relevant, we acknowledge its limitations. Our study has the main bias in its retrospective nature, which is partially overcome by the high number of cases included and by the presence of an electronic prospectively maintained database in each of the 2 high-volume institutes. In addition, there could be some differences in terms of the assessment of the clinical response to neoadjuvant treatments between the 2 centers. Especially at the beginning of the series, there could be some differences in the assessment of local endoluminal residual disease that, for example, during the digital examination, may be subjective. However, the majority of patients enrolled in our study also have been included in a large ongoing prospective observational multicenter trial.^29^

Our results showed that the actual criteria for defining a clinical response are suboptimal. Failure to achieve good sensitivity and precision represents a major limiting factor in the clinical setting.

## Supplementary Material


